# Seroepidemiology of *Toxoplasma gondii *infection in pregnant women in a public hospital in northern Mexico

**DOI:** 10.1186/1471-2334-6-113

**Published:** 2006-07-13

**Authors:** Cosme Alvarado-Esquivel, Antonio Sifuentes-Álvarez, Sergio Guadalupe Narro-Duarte, Sergio Estrada-Martínez, Juan Humberto Díaz-García, Oliver Liesenfeld, Sergio Arturo Martínez-García, Arturo Canales-Molina

**Affiliations:** 1 Faculty of Medicine, Juárez University of Durango State (UJED). Durango, Mexico; 2 General Hospital of Durango City, Secretary of Health. Durango, Mexico; 3 Institute for Scientific Research, UJED. Durango, Mexico; 4 Institute for Infection Medicine. Free University of Berlin, Germany

## Abstract

**Background:**

*Toxoplasma gondii *(*T. gondii*) infection in pregnant women represents a risk for congenital disease. There is scarce information about the epidemiology of *T. gondii *infection in pregnant women in Mexico. Therefore, we sought to determine the prevalence of *T. gondii *infection and associated socio-demographic, clinical and behavioural characteristics in a population of pregnant women of Durango City, Mexico.

**Methods:**

Three hundred and forty three women seeking prenatal care in a public hospital of Durango City in Mexico were examined for *T. gondii *infection. All women were tested for anti-*T. gondii *IgM and IgG antibodies by using IMx Toxo IgM and IMx Toxo IgG 2.0 kits (Abbott Laboratories, Abbott Park, IL, USA), respectively. Socio-demographic, clinical and behavioural characteristics from each participant were also obtained.

**Results:**

Twenty one out of the 343 (6.1%) women had IgG anti-*T. gondii *antibodies. None of the 343 women had IgM anti-*T. gondii *antibodies. Multivariate analysis using logic regression showed that *T. gondii *infection was associated with living in a house with soil floor (adjusted OR = 7.16; 95% CI: 1.39–36.84), residing outside of Durango State (adjusted OR = 4.25; 95% CI: 1.72–10.49), and turkey meat consumption (adjusted OR = 3.85; 95% CI: 1.30–11.44). Other characteristics as cat contact, gardening, and food preferences did not show any association with *T. gondii *infection.

**Conclusion:**

The prevalence of *T. gondii *infection in pregnant women of Durango City is low as compared with those reported in other regions of Mexico and the majority of other countries. Poor housing conditions as soil floors, residing in other Mexican States, and turkey meat consumption might contribute to acquire *T. gondii *infection.

## Background

*Toxoplasma gondii *(*T. gondii*) is a protozoan parasite widely distributed around the world [1,2]. It has been estimated that up to one third of the world's population is infected by *T. gondii *[3]. This parasite is transmitted to humans mainly by ingesting food or water that is contaminated with oocysts shed by cats or by eating undercooked or raw meat containing tissue cysts [3-5]. Infections in humans are usually asymptomatic but in some infected persons cervical lymphadenopathy or ocular disease may occur [1,3]. However, primary infection acquired during pregnancy may result in severe damage to the foetus [3,6]. Manifestations of congenital toxoplasmosis include mental retardation, seizures, blindness, and death [7]. Congenital disease may become apparent at birth or not until the second or third decade of life [7-9]. Acute and latent *T. gondii *infections during pregnancy are mostly diagnosed by serological tests including detection of anti-*T. gondii*-specific IgM and IgG antibodies [3,7-9], and avidity of *T. gondii*-specific IgG antibodies [3,10]. Reports of epidemiological studies indicate that prevalence of *T. gondii *infection in pregnant women varies substantially among countries. For instance, in European countries, prevalences of *T. gondii *infections in pregnant women vary from 9% to 67% [11-20]. In contrast, in Asian countries, low prevalences of *T. gondii *infection were found in a Korean study [21], and a Vietnamese study [22] (0.8% and 11.2%, respectively). While prevalences as high as 41.8% to 55.4% in pregnant women have been reported in Indian [23,24], Malaysian [25] and Nepalese [26] populations. For its part, a Sudanese study showed that 34.1% of the pregnant women studied had anti-*T. gondii *antibodies [27]. Similarly, a study performed in New Zealand revealed a 33% prevalence of anti-*T. gondii *antibodies [28]. In the American continent, a study performed in south Brazil revealed that 74.5% of the pregnant women studied had anti-*T. gondii *IgG antibodies [29]. In a Cuban study, 70.9% of women had anti-*T. gondii *antibodies 12 weeks before pregnancy [30]. There is scarce information about the epidemiology of *T. gondii *infection in pregnant women living in northern Mexico. Therefore, we performed a cross-sectional study in order to determine the prevalence of *T. gondii *infection in pregnant women of Durango City, Mexico and to know whether any characteristic of the women is associated with the infection.

## Methods

### Study population

All pregnant women seeking prenatal care from July 2005 to March 2006 at the General Hospital of Durango City, Mexico were invited to participate in the study. During the study period, 408 pregnant women were attended. Out of the 408 pregnant women, 343 were included in the study, and 65 were not included because either they did not accept to participate, or did not provide blood for analysis or did not submit the questionnaire. Inclusion criteria for the study subjects were: 1) pregnant women in any of the three trimesters of pregnancy; 2) aged 13 years and older; and 3) who accepted to participate in the study. Participants were enrolled consecutively.

### Socio-demographic, clinical and behavioural data

Socio-demographic data including age, birth place, residence place, marital status, occupation, educational level, socio-economic level and housing conditions index were obtained from all participants. Housing conditions index was obtained by using the Bronfman's criteria [31]. Briefly, five variables were evaluated: number of persons in the house, number of rooms in the house, material of the floor of the house, availability of drinkable water, and form of elimination of excretes. Clinical data including obstetric history, gestational age, blood transfusion or transplant history; and behavioural data including animal contacts, cleaning up cat excrement, foreign travel, kind of meat consumption (pork, lamb, beef, goat, boar, chicken, turkey, rabbit, deer, squirrel, horse, fish and iguana), raw or undercooked meat consumption, unpasteurized milk or milk products consumption, untreated water consumption, eating dried or cured meat (chorizo, ham, sausages or salami), unwashed raw vegetables or fruits consumption, contact with soil (gardening or agriculture), and eating outside of the home from all women studied were obtained.

### Laboratory tests

Sera of the pregnant women were analysed for anti-*T. gondii *IgM and IgG antibodies by IMx Toxo IgM and IMx Toxo IgG 2.0 kits (both from Abbott Laboratories, Abbott Park, IL, USA), respectively. These tests are based on Microparticle Enzyme Immunoassays technology and performed on the IMx immunoassay automated analyser (Abbott Diagnostics, North Chicago, Il, USA).

### Ethical aspects

This study was approved by the ethical committee of the General Hospital of Durango City. The purpose and procedures of the study were explained to all participants, and a written informed consent was obtained from all of them.

### Statistical analysis

Results were analyzed with the aid of the software Epi Info 6 and SPSS 8.0. For comparison of the frequencies among the groups, the Mantel-Haenszel test, and when indicated the Fisher exact test, were used. Bivariate and multivariate analyses were used to assess the association between the characteristics of the subjects and the *T. gondii *infection. Variables were included in the multivariate analysis if they had a p value of less than 0.2 in the bivariate analysis. Adjusted odd ratio (OR) and 95% confidence interval (CI) were calculated by multivariate analysis using a multiple, unconditional logic regression model.

## Results

### Sociodemographic description of the study population

General socio-demographic characteristics of the 343 pregnant women studied are shown in Table 1. The mean age of the pregnant women was 25 years (range: 13 to 44 years), and all studied women belonged to a low socio-economic level. Most women were born in Mexico and resided in urban areas of Durango State. The majority of them were housewives, have studied up to 12 years, and used to live in regular housing conditions. In addition, most women had had two or more pregnancies at the moment of the study.

**Table 1:** General sociodemographic characteristics of the pregnant women studied.

Characteristic	No. of women	%
Age groups (years)		
13–24	178	52.7
25–34	130	38.5
35–44	30	8.9
Birth place		
Mexico	342	99.7
USA	1	0.3
Residence State		
Durango State	334	97.7
Other Mexican State	8	2.3
Residence area		
Urban	263	77.6
Suburban	32	9.4
Rural	44	13
Educational level		
No education	13	4
Up to 12 years (College)	297	91.7
13–19 years (Undergraduate)	14	4.3
Occupation		
None	1	0.3
Student	21	6.2
Housewife	296	87.6
Employee	14	4.1
Professional	5	1.5
Business	1	0.3
Housing conditions index		
Bad	19	9.1
Regular	323	90.9
Trimester of pregnancy		
First	22	6.5
Second	80	23.6
Third	237	69.9
Number of pregnancies		
One	117	34.1
Two to four	192	56
Five or more	34	9.9

### Serology and prevalence

Out of the 343 pregnant women studied, twenty one were positive for anti-*T. gondii *IgG antibodies and none were positive for anti-*T. gondii *IgM antibodies. Therefore, we found a 6.1% prevalence of latent *T. gondii *infection, and 0% prevalence of acute *T. gondii *infection.

### Factors associated with seropositivity

In the bivariate analysis, seven variables were identified as possible risk factors associated with *T. gondii *infection: 1) living in a house with soil floors (p = 0.004); 2) a residence outside Durango State (p = 0.009); 3) turkey meat consumption (p = 0.06); 4) deer meat consumption (p = 0.08); 5) squirrel meat consumption (p = 0.1); 6) wild animals meat consumption (p = 0.06); and 7) blood transfusion (p = 0.1). The rest of the sociodemographic, clinical and behavioural characteristics of the studied women did not show any likely association with *T. gondii *infection. Table 2 shows the results of the bivariate analysis of selected variables and the results of *T. gondii *seropositivity. As seen in Table 3, by using multivariate analysis, only three variables were associated with *T. gondii *seropositivity: 1) living in a house with soil floor (adjusted OR = 7.16; 95% CI: 1.39–36.84); 2) residing outside Durango State (adjusted OR = 4.25; 95% CI: 1.72–10.49); and 3) turkey meat consumption (adjusted OR = 3.85; 95% CI: 1.30–11.44).

**Table 2:** Bivariate analysis of selected characteristics of the pregnant women studied and *T. gondii *infection.

Characteristic	Pregnant women^a^	Positive test for anti-*T gondii *antibodies	P value
	No. 343 (100%)	No. 21 (6.1%)	
Pork meat consumption			
Yes	308 (90.1)	20 (6.5)	0.3
No	34 (9.9)	1 (2.9)	
Beef meat consumption			
Yes	337 (98.3)	21 (6.2)	0.6
No	6 (1.7)	0 (0)	
Turkey meat consumption			
Yes	102 (29.7)	10 (9.8)	0.06
No	241 (70.3)	11 (4.6)	
Deer meat consumption			
Yes	18 (5.2)	3 (16.7)	0.08
No	325 (94.8)	18 (5.5)	
Squirrel meat consumption			
Yes	12 (3.5)	2 (16.7)	0.1
No	331 (96.5)	19 (5.7)	
Wild animal meat comsumption^b^			
Yes	26 (7.6)	4 (15.4)	0.06
No	315 (92.4)	17 (5.4)	
Degree of meat cooking			
Raw or undercooked	7 (1.7)	0 (0)	0.6
Well done	401 (98.3)	21 (5.2)	
Unwashed raw vegetable or fruit consumption			
Yes	228 (66.5)	13 (5.7)	0.6
No	115 (33.5)	8 (7)	
Contact with cat^c^			
Yes	286 (83.4)	17 (5.9)	0.4
No	57 (16.6)	4 (7)	
Gardening or agriculture			
Yes	186 (54.2)	13 (7)	0.4
No	157 (45.8)	8 (5.1)	
Residence outside Durango			
Yes	8 (2.3)	3 (37.5)	0.009
No	334 (97.7)	18 (5.4)	
Blood transfusion			
Yes	24 (7.1)	3 (12.5)	0.1
No	316 (92.9)	18 (5.7)	
Soil floors			
Yes	28 (8.3)	6 (21.4)	0.004
No	311 (91.7)	15 (4.8)	

^a^Women with available data.

^b^Including deer, boar or squirrel.

^c^Cat in home or neigborhood or cleaning cat feces.

**Table 3:** Multivariate analysis of characteristics of the pregnant women studied and their association with *T. gondii *infection.

Characteristic^a^	Group	Adjusted odds ratio	95% Confidence interval	P value
Turkey meat consumption				
	Yes	3.85^b^	1.30–11.44	0.014
	No			
Deer meat consumption				
	Yes	0.8^b^	0.04–16.47	0.9
	No			
Squirrel meat consumption				
	Yes	1.38^b^	0.10–18.95	0.8
	No			
Wild animal meat comsumption^c^				
	Yes	2.4^b^	0.09–61.01	0.5
	No			
Residence outside Durango				
	Yes	4.25^b^	1.72–10.49	0.001
	No			
Blood transfusion				
	Yes	28.9^b^	0.00–250	0.8
	No			
House with soil floors				
	Yes	7.16^b^	1.39–36.84	0.018
	No			

^a^The variables included were those with a p < 0.20 obtained in the bivariate analysis.

^b^Adjusted by age, occupation, housing conditions index, contact with cat and the rest of Characteristics included in this table.

^c^Including deer, boar or squirrel.

## Discussion

In spite of about 2 cases of congenital toxoplasmosis per 1000 newborns occur in Mexico City [32], the epidemiology of *T. gondii *infection in general, and in pregnant women in particular, have been poorly studied in Mexico. A national survey in the general population showed that *T. gondii *infections exist in the whole country [33], and prevalences clearly vary among the regions [33,34]. With respect to research on *T. gondii *infection in pregnant women of Mexico, only three studies have been reported. A study performed in a humid tropical State located in the south-east of Mexico showed that 60% of the pregnant women studied were positive for anti-*T. gondii *antibodies [35]. Other study performed with high risk pregnant women from central Mexico showed that 34.9% had anti-*T. gondii *IgG antibodies [36], and a study of women with spontaneous abortions from the south of Mexico showed a 47% prevalence of anti-*T. gondii *antibodies [37]. In this study, we found a 6.1% prevalence of latent *T. gondii *infection in pregnant women of Durango City, Mexico. This prevalence is much lower than those reported in the previous studies of pregnant women in other regions of Mexico [35-37]. Similarly, our prevalence found in Durango city is much lower than those reported in pregnant women from European countries [11-20], Vietnam [22], India [23,24], Malaysia [25], Nepal [26], Sudan [27], New Zealand [28], Brazil [29], and Cuba [30], where prevalences vary from 9% to 74.5%. In contrast, our prevalence is higher than the one reported in a Korean study, where researchers found a prevalence of about 0.8% in the pregnant women studied [22]. The lower prevalence found in our study compared with those reported elsewhere might be explained by differences in the characteristics of the pregnant women studied and the environment of Durango City. Concerning the characteristics of the pregnant women that may contribute to the low prevalence found could be: 1) the pregnant women studied belonged to a low socio-economic level and although they do eat meat, they certainly do it at a lower frequency and quantity than those with a better socioeconomic level because they can not afford to buy meat in a regular basis. Since eating contaminated meat is a well known route of *T. gondii *infection, the lower the frequency of meat consumption the lower the risk of infection; and 2) consumption of undercooked or raw meat is a rare practice both in the studied women and in people of the general population of Durango City. The presence of these two characteristics in our infected population points towards transmission that might have occurred in some cases, not by ingesting tissue cysts of the parasite in infected meat but by ingesting parasite oocysts in contaminated food or water. The poorest housing conditions found among the infected population support this statement. With respect to environmental characteristics, we may speculate that the low prevalence found could be also explained by a number of environment characteristics: 1) Durango City has a dry climate, and prevalence of *T. gondii *infection in dry climates has been reported lower than other climates [3,19,33]; 2) Most of the year Durango City usually has high temperatures during the day, and these high temperatures may contribute to reduce infectivity of *T. gondii *oocysts. This likely explanation could be supported by the observation in an experimental model that the higher the storing temperature of *T. gondii *oocysts the shorter the survival and infectivity time of the parasites [38]; and 3) Durango City also has a high altitude (1880 meters above sea level), and prevalences of *T. gondii *infection have been found lower in high altitudes than in low altitudes in some studies in humans [39,40] and a study in animals [41].

In this study, living in a house with soil floors was associated with *T. gondii *infection (adjusted OR = 7.16; 95% CI: 1.39–36.84). Contaminated soil with parasite oocysts is a source of infection [6] and might contribute to explain the higher frequency of infection in women living in a house with floors made of soil than those living in a house with floors made of concrete or other materials. Theoretically, infected cats play a major role in contaminating soil; therefore persons living in a house with soil floors need to have a cat or have contact with it to become infected by this route. The fact that in this study the variable "contact with cats" was not associated with infection might be explained by a number of possible reasons: 1) contact with cats was a very common practice among the studied population, but parasite transmission could be more efficient in those houses with soil floors than in those with other kind of floors; 2) the frequency of infection in cats may be higher in those living in the poorest environment than those living in better housing conditions. The food consumed by the cats is certainly different in those living in the poorest housing condition than those living in a regular housing condition. More hunting of possibly infected animals may occur in the poorest environment than in a better one; 3) soil floors reflect extreme poverty and there are always further bad sanitary conditions in women with this status that may all facilitate transmission of *T. gondii*, and; 4) animals other than domestic cats may also contaminate soil floors. In this study we also found an association of latent *T. gondii *infection with a residence outside Durango State (adjusted OR = 4.25; 95% CI: 1.72–10.49). This finding suggests that those women could have been infected outside Durango State. In addition, logistic regression showed that turkey meat consumption was also associated with *T. gondii *infection (adjusted OR = 3.85; 95% CI: 1.30–11.44). Turkey meat consumption could be thus responsible for at least some cases of *T. gondii *infection among our infected population. This finding was unexpected since meats other than turkey meat have been implicated more frequently as a source of *T. gondii *infection than turkey meat. This finding deserves further investigation. The rest of the sociodemographic, clinical, and behavioural characteristics of the studied women did not show any association with *T. gondii *infection. Known factors associated with *T. gondii *infection in pregnant women such as drinking untreated water [13,42], contact with garden soil [29], increasing parity [25], consumption of cured meat [16], and contact with cats [43], were not associated with *T. gondii *infection in our study.

We performed serum analysis by Microparticle Enzyme Immunoassays for anti-*T. gondii *IgG and IgM antibodies because these assays have been shown a high overall agreement as compared with the dye test [44]. In addition, routine quality control measure were applied when analysing samples, therefore, we considered results of our assays were reliable.

## Conclusion

We concluded that the prevalence of *T. gondii *infection in pregnant women of Durango City is low as compared with those reported in other regions of Mexico and the majority of other countries. Poor housing conditions as soil floors, residing in other Mexican States, and turkey meat consumption might contribute to acquire *T. gondii *infection.

## Competing interests

The author(s) declare that they have no competing interests.

## Authors' contributions

CAE conceived and designed the study protocol, participated in the coordination and management of the study, performed the data analysis and wrote the manuscript. ASA designed the study protocol, applied the questionnaires and performed the data analysis. SGND performed the analysis of the serum samples. SEM performed the statistical analysis. JHDG applied the questionnaires and performed the data analysis. OL designed the study protocol, performed the data analysis, and wrote the manuscript. SAMG performed the data analysis. ACM applied the questionnaires and monitored the study.

## Pre-publication history

The pre-publication history for this paper can be accessed here:


